# Research Progress of NiMn Layered Double Hydroxides for Supercapacitors: A Review

**DOI:** 10.3390/nano8100747

**Published:** 2018-09-20

**Authors:** Ai-Lan Yan, Xin-Chang Wang, Ji-Peng Cheng

**Affiliations:** 1 Institute of Hydraulic and Environmental Engineering, Zhejiang University of Water Resources and Electric Power, Hangzhou 310018, China; yal200@126.com; 2 Key Laboratory of Material Physics, School of Physics and Engineering, Zhengzhou University, Zhengzhou 450052, China; wxclhm@zzu.edu.cn; 3 State Key Laboratory of Silicon Materials, School of Materials Science and Engineering, Zhejiang University, Hangzhou 310027, China

**Keywords:** NiMn layered double hydroxide, electrode materials, hydrotalcite, supercapacitor, composite, asymmetric capacitor

## Abstract

The research on supercapacitors has been attractive due to their large power density, fast charge/discharge speed and long lifespan. The electrode materials for supercapacitors are thus intensively investigated to improve the electrochemical performances. Various transition metal layered double hydroxides (LDHs) with a hydrotalcite-like structure have been developed to be promising electrode materials. Earth-abundant metal hydroxides are very suitable electrode materials due to the low cost and high specific capacity. This is a review paper on NiMn LDHs for supercapacitor application. We focus particularly on the recent published papers using NiMn LDHs as electrode materials for supercapacitors. The preparation methods for NiMn LDHs are introduced first. Then, the structural design and chemical modification of NiMn LDH materials, as well as the composites and films derived from NiMn LDHs are discussed. These approaches are proven to be effective to enhance the performance of supercapacitor. Finally, the reports related to NiMn LDH-based asymmetric supercapacitors are summarized. A brief discussion of the future development of NiMn LDHs is also provided.

## 1. Introduction

In recent years, two-dimensional (2D) layered inorganic materials have attracted much interest in various application fields of novel composites, energy storage, biomedicine, catalysis*,* etc. [1,2,3,4]. There are many types of layered inorganic materials and they can be grouped into diverse families, including hexagonal graphene, hexagonal boron nitride, metal dichalcogenides, metal halides, layered double hydroxides, etc. [1]. Layered double hydroxide (LDH), also known as a hydrotalcite-like compound, is one of the most widely studied layered inorganic materials and has been applied in many fields [4,5,6]. LDH crystals are composed of positively charged hydroxide host layers, intercalated anions and water molecules. The hydroxyl groups in the host layers are oriented toward the interlayer space and usually are hydrogen bonded to these intercalated anions and water molecules [7]. LDH materials have a weak interlayer bonding force and a large interlayer distance, and exhibit excellent exfoliation properties. LDHs can be exfoliated into ultrathin nanosheets in special solvents due to the exchangeable anions in the interlayer space to neutralize the positively charged host layers, which will facilitate the preparation of monolayer blocks [8]. Meanwhile, LDHs can be easily synthesized by some well-established synthesis protocols including co-precipitation and hydrothermal method. They can simply be prepared in a large scale with a low cost for industrial application [9,10].

Nowadays, the research for novel and sustainable energy storage technologies has been intensively carried out. Among the many energy storage devices, supercapacitors (SCs), also called electrochemical capacitors, have obtained significant attention from researchers worldwide. SC is an important energy storage device due to its large power density, ultrafast charge/discharge rate, and long life. The electrochemical performances of a SC are essentially determined by the properties of electrode materials, including the chemical composition, morphology, and microstructure [11]. There are generally two kinds of SCs, i.e., electrical double-layered capacitor (EDLC) and pseudocapacitor, according to their different work mechanisms. EDLC is based on the electrostatic storage on the interface of electrode materials and electrolyte, typically for the carbon materials with high surface areas. The other relies on reversible Faradaic reactions that occur on the surface of electrode materials such as metal oxides, metal hydroxide, metal sulfides, etc. [2].

The biggest bottleneck that limits the application of SCs is its low energy density. To overcome this disadvantage, research on electrode materials is one of the most important issues [12]. Transition metal-based LDHs have been considered as potential electrode materials due to their high redox activities, natural abundance and unique structure [13]. The easy tunability of the metal cations in the host layers and the exchangeability of anion in the interlayer space without influencing the structure endow them with many intriguing electrochemical properties. Meanwhile, some LDHs can be exfoliated into monolayer nanosheets in some specific solvents, even in water [14]. Thus, there have been a lot of research on the electrochemical behavior of LDHs for SC application [12].

Some review papers related to LDHs for SC application have been published in recent years [3,12,15,16,17,18,19]. NiCo LDHs, CoAl LDHs, CoFe LDHs, NiAl LDHs, NiCoAl LDHs, CoMn LDHs, NiMn LDHs, etc. are popularly reported as electrode materials for SCs, among which NiCo LDH materials have received the most intensive studies. α-Ni(OH)_2_ is also an anionic clay with a hydrotalcite-like structure and it can exchange the interlayer anions from the alkaline electrolyte. It has been studied as a perspective electrode material for SC. The partial substitution of Ni(II) with some trivalent cations (e.g., Al, Fe, Co, Cr, and Mn) in the host layers can stabilize the structure of α-Ni(OH)_2_ due to the increase of the positively charged host layers [20]. Thanks for the variable oxidation states from Mn, it has also been used as an important component of batteries electrode material [21,22]. The substitution of Ni(II) with Mn(III) species will lead to high dispersion of Ni in the host layers, then boosting the utilization of Ni species. Thus, the research on NiMn LDHs for SCs application has drawn much attention recently and some reports on this topic have been published. In this paper, we mainly focus on the recent investigation of NiMn-based LDHs for the application of SCs. As far as we know, this is the first review on the latest progress in the nanostructured NiMn LDHs for SCs. NiMn LDH and its composites for SC application have been discussed in this review. The asymmetric CSs based on NiMn LDH electrode are considered. The prospects of NiMn LDH and its composites are also presented in this paper.

## 2. The Synthesis Method of NiMn LDHs

Compared to other 2D layered materials, LDHs can be easily prepared by conventional wet-chemical methods, including co-precipitation of metal salts in alkaline solution, hydrothermal process and ion-exchange method. LDHs containing earth abundant metals represent promising electrode materials due to their unbeatable advantages, such as low cost and abundant availability, as well as competent electrochemical activity and durability. For NiMn LDHs, Ni and Mn are two earth abundant elementals with high electrochemical activity and can also be easily prepared by facile method. In this section, the preparation method for NiMn LDHs is reviewed first.

The most popular and oldest method to prepare NiMn LDHs is co-precipitation, where dissolvable Ni and Mn salts are reacted with some basic chemicals under rather low temperatures in aqueous solutions. Ammonia solution [23,24], NaOH and Na_2_CO_3_ [25,26], LiOH [27], hexamethylenetetramine [28] and urea [29] can be used as basic chemicals to produce hydroxide anions. 

Both urea and hexamethylenetetramine can act as hydrolysis agents to slowly alkalize aqueous solution and homogeneously precipitate out NiMn LDHs at a moderate temperature, usually below 90 °C. Thus, the reaction system is usually heated and maintained under a moderate temperature to keep the reaction occurring. Guo et al. showed a core/shell design using porous NiCo_2_O_4_ nanowires as core and NiMn LDH nanosheets as shell by co-precipitation method using urea at 80 °C for 80 min [29]. They also applied the same method to deposit NiMn LDH onto the surface of KCu_7_S_4_ microwires with urea at 80 °C for 2 h [30]. Zhao et al. reported that NiMn LDHs were prepared by the reaction of hexamethylenetetramine with metal salts at 90 °C under stirring for 4 h [28]. 

Due to the mild reaction conditions, NiMn LDHs even can be prepared at room temperature by using some typical chemicals with a low energy consumption. Barriga et al. [31] reported that the synthesis of NiMn LDHs with different Ni/Mn ratios was performed by dropwise addition of a 100 mL solution containing Ni(NO_3_)_2_ and MnCl_2_ to 100 mL solution of 0.2 M NaOH and 0.1 M Na_2_CO_3_ at room temperature under an air atmosphere, and that Mn(II) was partially oxidized to be Mn(III) during the synthesis process. More recently, Zhou reported that the solution of Ni(NO_3_)_2_ and Mn(NO_3_)_2_ could be reacted with the mixture solution of NaOH and Na_2_CO_3_ at room temperature for 12 h to produce NiMn LDHs [25]. To accelerate the oxidation of Mn^2+^, oxygen bubbles or H_2_O_2_ were added to the reaction solution throughout the experimental process [26]. Li et al. reported that ammonia solution could react with Ni(II) and Mn(II) salts to deposit NiMn LDH onto the surface of carbon materials at room temperature [23].

In addition to co-precipitation in aqueous solutions, some special solvents, such as methanol and ethanol, are also applied to prepare NiMn LDHs. Latorre-Sanchez et al. [32] reported the synthesis of NiMn LDHs in methanol, where Ni/Mn salts and NaOH were reacted in methanol at 65 °C for three days with stirring to obtain a NiMn LDH methanolic suspension. Then, an exfoliated NiMn LDHs aqueous suspension could be prepared by diluting the methanolic suspension in water and sonication. Li et al. prepared NiMn LDHs on the surface of spherical SiO_2_ to fabricate hollow NiMn LDH microspheres, where the reaction was performed in ethanol at 50 °C for 1 h [33].

Hydrothermal method is also popularly applied to synthesize NiMn LDHs due to its high efficiency. However, the reaction temperature of hydrothermal method is usually higher than that of co-precipitation. These basic chemicals used in co-precipitation are also effective for hydrothermal method. Although NaOH can be quickly reacted with metal ions to produce NiMn LDHs [34], urea is one of the ideal chemicals to produce LDHs during hydrothermal process [35]. Well-defined NiMn LDH nanoplates can be easily fabricated by urea (or hexamethylenetetramine) hydrolysis, which takes the advantage of the phenomenon that urea (or hexamethylenetetramine) aqueous solution is neutral at low temperature but hydrolyzes above 90 °C to realize a basic solution. The homogenous increase of pH value by urea (or hexamethylenetetramine) hydrolysis can facilitate the preparation of LDHs with high crystallinity under some controllable conditions. Yan et al. prepared NiMn LDH nanoarrays on melamine sponge-derived carbon by hydrothermal method [35]. Meanwhile, hexamethylenetetramine could also be used to prepare NiMn LDHs grown on Ni foam by hydrothermal process at 90 °C for 6 h [36]. In addition to the reactions in aqueous solutions, a mixture solution containing deionized water and ethanol can be used, as reported by Huang et al. [37]. Similar to co-precipitation method, Chang et al. found that ammonium peroxodisulfate oxidized Mn^2+^ ions into Mn^3+^ ones during the hydrothermal process and it played an important role for the generation of Ni(II)Mn(III) CO_3_^2−^ LDHs [38]. Singh et al. [39] reported that cetyltrimethylammonium bromide could react with metal ions to generate NiMn LDHs and cobalt-doped NiMn LDHs on Ni foam. Thus, these basic chemicals that can hydrolyze under a moderate temperature are more commonly applied in hydrothermal method than in co-precipitation process, because hydrothermal process usually occurs under a high temperature.

**Figure 1:**
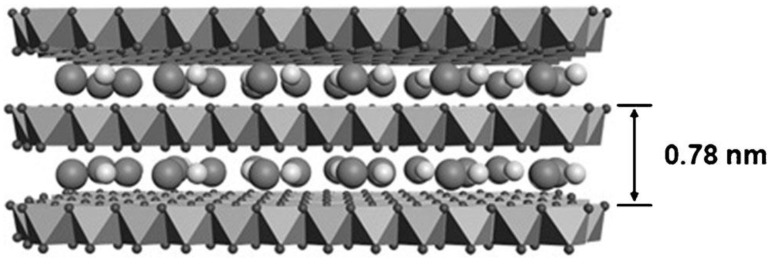
The structure for NiMn LDH nanosheets. Reproduced with permission from [40]. Copyright Wiley, 2014.

In addition to the two common methods above, Sim et al. [40] reported a reverse micelle method to prepare colloidal NiMn LDH nanosheets with a well-defined morphology. In the process, xylene was used as an oil phase. Oleylamine and oleic acid were employed as surfactant and co-surfactant, respectively. Their results suggested that the NiMn LDHs nanosheets had a formula of [Ni_3_Mn(OH)_8_](Cl^−^)·nH_2_O, as depicted in Figure 1. The interlayer distance is about 0.78 nm. In this method, some toxic solvents are involved.

## 3. Electrochemical Performances of NiMn LDH-Based Electrode Materials

### 3.1. Energy Storage Mechanism for SCs

NiMn LDHs can be dissolved in acidic aqueous solutions. Thus, some alkaline medium, such as KOH, NaOH, and LiOH solution, is selected as electrolyte for SCs. NiMn LDHs have the Faradaic redox reactions during the energy storage process, which can be expressed as follows

Ni(OH)_2_ + OH^−^ ⇔ NiOOH + H_2_O + e^−^ (1)

Mn(OH)_2_ + OH^−^ ⇔ MnOOH + H_2_O + e^−^ (2)

MnOOH + OH^−^ ⇔ MnO_2_ + H_2_O + e^−^ (3)

Charge transportation happens through the NiMn LDHs host layers via electron hopping between the redox sites and ion motion across the pores or channels in the electrode material to keep the electroneutrality of LDH structure [41]. The effect of the scan rate (ν) on the electrochemical response of NiMn LDH exhibits that the redox reaction is governed by a diffusion-controlled process, which can be indicated from the linear relationship between ν^1/2^ and peak current from both redox peaks.

### 3.2. Designing and Chemical Modification of NiMn LDHs

Although most transition metal LDHs can be used as promising electrode materials, the Faradaic reactions during the charging–discharging processes are dramatically limited by the low electrical conductivity and slow electrolyte penetration. To overcome these disadvantages, some strategies have been developed to enhance the electrochemical performances, such as combining LDH with highly conductive nanomaterials to form composites, constructing reasonably porous LDH nanostructures with high surface areas, and exfoliation or delamination of LDHs into single-layer nanosheets. For NiMn LDHs, the aforementioned approaches are all effective for SC application.

**Figure 2:**
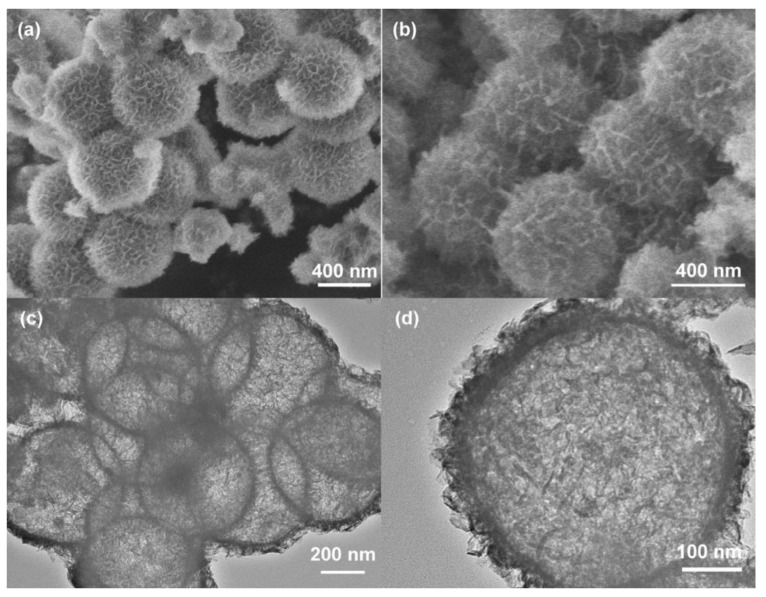
SEM images of: (**a**) SiO_2_@NiMn LDHs; and (**b**) NiMn LDHs hollow spheres. (**c**,**d**) TEM images of NiMn LDHs hollow spheres. Reproduced with permission from [33]. Copyright Elsevier, 2017.

For pure NiMn LDH material, the surface area and pore volume are two key features to influence its electrochemical performance. NiMn LDH material with a porous nanostructure usually exhibits a better performance than that having a conventional structure. Porous nanostructures of NiMn LDH provide electrons and ions short transport path lengths, resulting in fast kinetics and more active centers for surface Faradaic reactions. Li et al. [33] applied SiO_2_ spheres as sacrifice cores to deposit NiMn LDHs nanosheets as exterior shells. After removal of SiO_2_ spheres, hollow NiMn LDH spheres with high electrochemical performances were obtained, as shown in Figure 2. The hollow NiMn LDHs delivered a specific capacitance of 595.6 F g^−1^ at 1 A g^−1^ and a good stability. 

More recently, Zhou et al. [25] constructed a hierarchical structure by deposition of NiMn LDHs and poly(3,4-ethylenedioxythiophene) on halloysite nanotubes via growth and polymerization. The composite material exhibited a 3D architecture having a high surface area and well-defined core–shell structure. The NiMn LDHs/halloysite electrode materials delivered a specific capacitance of 1665.6 F g^−1^ at 1 A g^−1^. However, the halloysite core had little contribution to the energy storage in this composite, but to disperse LDH uniformly on its surface. Guo and co-workers reported an approach to prepare NiMn LDH arrays on KCu_7_S_4_ microwires via heterogeneous nucleation growth [30]. Quasi-paralleled and interpenetrated NiMn LDHs arrays on KCu_7_S_4_ microwires were controlled during the fabrication process. The KCu_7_S_4_@NiMn LDHs composite had a high specific capacitance (733.8 F g^−1^ at 1 A g^−1^) in 1 M LiOH electrolyte.

The interlayer gallery of metal LDHs contains additional anions, such as nitrate, carbonate, or chloride, which act as charge-balancing anions. The intercalated anions are usually exchangeable for LDHs, thus making it possible to change the interlayer distance. Previous reports proved that the intercalated anions and interlayer distance both had influence on the performance of LDHs [42,43]. Glucose intercalated NiMn LDH was synthesized by a one-pot hydrothermal method, as reported by Lv et al. [44], which expanded the interlayer distance to improve the cycling stability. Electrochemical tests showed that annealing-treated glucose intercalated NiMn LDH could deliver a specific capacity of 1464 F g^−1^ at 0.5 A g^−1^, much higher than pristine NiMn LDH.

The exfoliation property is an important feature for LDHs, implying an easy method to obtain ultrathin 2D building blocks for composite fabrication. The exfoliation of LDHs into many nanosheets with ultrathin thickness (<1 nm) can result in a high specific surface area and expose all metal sites [14,45], which will remarkably enhance the electrochemical performance. The exfoliated LDH nanosheets had an electrocatalytic efficiency that was even comparable to noble metal catalysts [46]. Ma et al. [46] prepared chloride anions intercalated NiMn LDHs for the first time by precipitation. After ion-exchange and exfoliation, NiMn LDHs nanosheets were flocculated together with reduced graphene oxide (rGO) to form a superlattice-like composite. The composite showed a low overpotential and a small Tafel slope for oxygen evolution reaction. Quan [47] reported a new pseudocapacitive nanocomposite and such composites were fabricated via restacking of NiMn LDH and MnO_2_ nanosheet suspensions that were obtained by exfoliation treatment. The composite had improved surface area and pore size, when compared with each component, either raw materials or simple mixture. However, research on exfoliated pure NiMn LDHs for SCs has been not reported yet.

### 3.3. NiMn LDHs Composites

Recently, the research for hierarchical or mixed composites consisting of LDHs and carbon materials, such as graphene, carbon nanotubes (CNTs), and rGO, has been rapidly developed to improve the electrical conductivity of NiMn LDH composites. In particular, graphene and CNTs have attracted much attention owing to their large surface area, excellent electrical conductivity and high mechanical strength. For carbon materials, the capacitance primarily comes from an electrical double-layer mechanism without the Faradaic reactions from NiMn LDH. For example, Huang et al. simply compared the electrochemical performances of NiMn LDH/rGO composites with different rGO contents, where the composites exhibited higher specific capacitance and better rate capability than pristine NiMn LDH [37]. In this section, we review the reports on NiMn LDH composites for the electrode materials of SCs.

**Figure 3:**
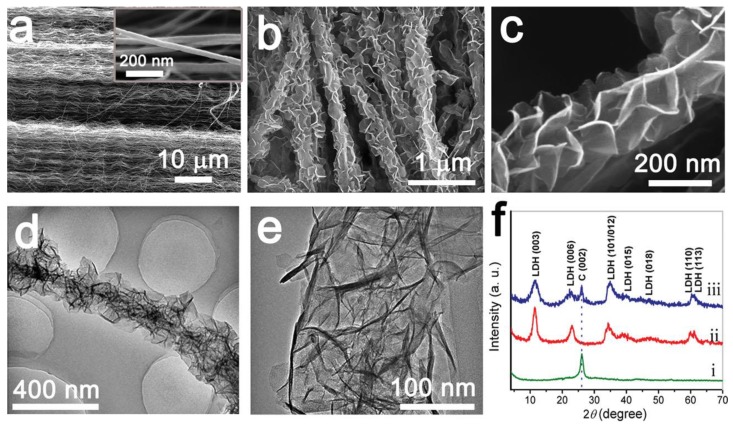
(**a**) SEM images of pristine CNTs; (**b**,**c**) SEM images NiMn LDH/CNTs; (**d**,**e**) TEM images of the composite NiMn LDH/CNTs; and (**f**) XRD patterns of CNTs (i), NiMn LDH (ii), and NiMn LDH/CNTs (iii). Reproduced with permission from [48]. Copyright Wiley, 2014.

Zhao et al. [48] firstly fabricated a hierarchical structure composed of many NiMn LDHs crystals anchored on the surface of CNTs constructed by an in situ growth route. The composites displayed a 3D structure with tunable Ni/Mn ratios, well-defined core–shell architecture, and high specific surface areas. The NiMn LDH/CNTs electrode with a Ni/Mn ratio of 3 was very active, which delivered the highest specific capacitance of 2960 F g^−1^ at 1.5 A g^−1^. The SEM images of CNTs and the resultant composite, as well as their TEM images are shown in Figure 3a–e. Meanwhile, the XRD patterns of the products are also exhibited in Figure 3f. The core–shell heterostructure of NiMn LDH/CNT can be clearly observed in both SEM and TEM images. The NiMn LDHs as shell acted electrochemically active species, while CNTs were used as both electron collector and LDH support.

In addition to CNTs, 2D graphene and rGO are both reported to combine with NiMn LDHs in order to prepare high performance composite materials. Padmini et al. [49] prepared the composites consisting of NiMn LDH and rGO with different contents of rGO by a chemical solution method. The electrochemical performances of the NiMn LDH/rGO composites were measured in KOH solution in a three-electrode system. The composite of NiMn LDHs/rGO with 5% rGO showed the highest electrochemical activity, when compared to other composites with different rGO contents, pristine NiMn LDH and rGO. The enhanced electrochemical activity was mainly attributed to the large surface area and the high electrical conductivity of the composite. Lee et al. [50] used graphene as a substrate that was exfoliated from bulk graphite by ionic surfactants without using any strong acids and toxic reducing agents to support NiMn LDH. The specific capacitance of NiMn LDH was 2219 F g^−1^ at 0.73 A g^−1^. Above reports have demonstrated that NiMn LDH/graphene (or rGO) composites can improve not only the specific capacitance but also the cycling stability.

Porous carbon is also used to support NiMn LDH to fabricate advanced electrode materials. Yu et al. [51] applied zeolitic imidazolate frameworks-8 as the precursor of porous carbon to deposit NiMn LDH, and they obtained a polyhedral-like composite. The electrochemical performance of the composite was enhanced and it delivered a specific capacitance of 1634 F g^−1^ at 1 A g^−1^. They attributed the improved performance to the Faradaic pseudocapacitance from NiMn LDH, the presence of porous carbon as a core, as well as the 3D porous structure of composites.

**Figure 4:**
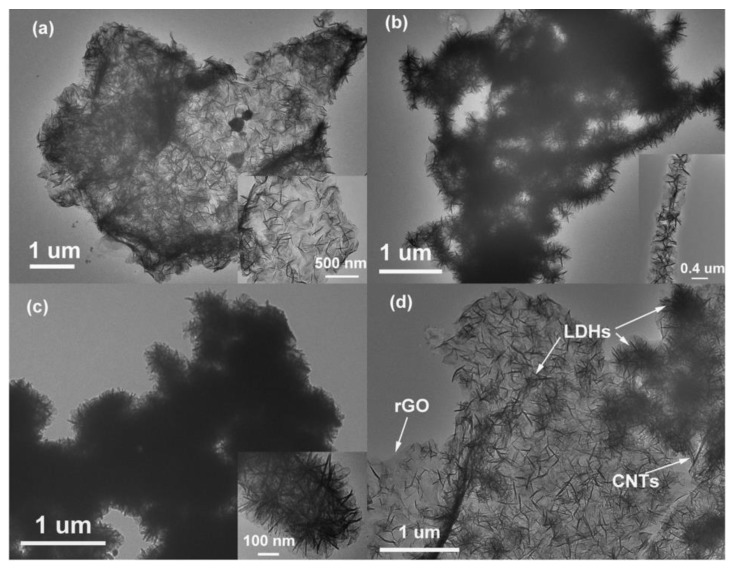
TEM images of: (**a**) NiMn LDH/rGO; (**b**) NiMn LDH/CNTs; (**c**) NiMn LDH/carbon black; and (**d**) NiMn LDH/CNTs/rGO [23]. Reproduced with permission from [23]. Copyright Royal Society of Chemisty, 2016.

Li et al. [23] applied three carbon nanomaterials from 0D carbon black particle, 1D CNTs to 2D rGO to in situ combine with NiMn LDH for SCs. Some typical TEM images of the composites are shown in Figure 4. The surface of rGO in the LDH/rGO composite is fully covered by thin laminar NiMn LDHs in Figure 4a. For LDH/CNTs composite, 1D CNTs are encircled by LDHs, as exhibited in Figure 4b. The flower-like NiMn LDH/carbon black particles are closely stacked together, as shown in Figure 4c. Figure 4d shows the ternary LDH/CNTs/rGO composite. A comparative study was performed on the composites of NiMn LDH/rGO, LDH/carbon black, LDH/CNTs and ternary LDH/CNTs/rGO. The results showed that ternary NiMn LDH/CNTs/rGO composite yielded the highest specific capacitance of 1268 F g^−1^ at 1 A g^−1^ in 2 M KOH and a long lifespan, exhibiting great potential for SC application. The ternary LDH/CNTs/rGO composite had a much higher specific surface area than those of LDH/CNTs and LDH/rGO. At the same time, Li et al. [23] found that the cations and concentrations of electrolyte influenced the performance of the composite. A high concentration of the alkaline electrolyte could enhance the electrochemical performance and aqueous NaOH showed many advantages for the composite due to the low resistance [23].

The aforementioned NiMn LDH composites are mostly powder product. Before a work electrode is prepared, the powder electrode material should be fully blended with some polymer binder and conductive additive in special solvent to paint them onto a current collector. In the above section, we mainly discuss the powder composites comprised of NiMn LDHs and high conductive materials. Meanwhile, NiMn LDH films can deposit onto a variety of bulky conductive substrates, such as carbon cloth and nickel foam, then to be used as an electrode directly. However, this increases the complexity of the synthetic procedures. NiMn LDH film on current collector can be used as a work electrode directly without involving organic binder, thus increasing the electrical conductivity and interface area between electrolytes and electrodes. In the following section, we review the previous reports on NiMn LDH films grown on the conductive substrates.

### 3.4. NiMn LDHs Film Electrodes

NiMn LDH crystals can be grown on some typical conductive substrates and serve as a work electrode directly. Nickel foam has been widely used as a substrate for electrode materials due to its low resistance, high surface area and 3D porous structure. It is generally applied as both deposition substrate and current collector for NiMn LDH films. Guo et al. [52] prepared aligned hierarchical NiMn LDH crystals on nickel foam by a one-step method and the as-prepared NiMn LDH@nickel foam presented oriented layered structure as very thin nanosheets. The optimized electrode could be prepared by fine tuning the mole ratio of Ni/Mn and displayed a high specific capacitance of 1511 F g^−1^ at 2.5 A g^−1^.

Some highly conductive building blocks, such as NiO, metal sulfides and graphene, pre-coated Ni foam are more attractive than Ni foam itself to deposit NiMn LDH, but the preparation process is somewhat complex. Liu et al. [53] applied a two-step hydrothermal method to prepare a composite of NiO/NiMn LDH nanosheet array on nickel foam to integrate the properties of each building block. NiO nanosheet arrays were prepared on nickel foam by hydrothermal method at first, and then NiMn LDH nanoarrays were deposited onto the surface of NiO arrays. Compared with the NiO and NiMn LDH electrodes on Ni foam prepared by the same method, the NiO/NiMn LDH composite electrode exhibited a much better electrochemical performance, about 510 F g^−1^ at 1 A g^−1^ in specific capacitance. The hierarchical-structured composite film can improve performances, because the film has highly exposed surface area with high accessibility for electrolyte [54]. Chen et al. [36] reported that 3D hierarchical carbon-coated NiMn LDH on Ni foam prepared via a two-step hydrothermal reaction could be used as a flexible electrode directly. The resulting composite had a specific capacitance of 1863 F g^−1^ at 1 A g^−1^ and an excellent cycling stability. Guo et al. [55] applied aligned graphene on Ni foam as a current collector to deposit NiMn LDHs subsequently, where the vertically aligned graphene nanosheets could promote the mass transport and further boost the electrochemical kinetics process. The stack density of NiMn LDH crystals related to the hydrophilicity of the composite films could be easily controlled by changing the feeding mole ratio of Ni to Mn. The as-prepared NiMn LDH-graphene@nickel foam hybrid with a 3:1 mole ratio of Ni to Mn showed a high specific capacitance of 2920 F g^−1^ at 2 A g^−1^. 

More recently, nickel foam coated by metal sulfide nanorods has received much attention for depositing LDHs. Yu et al. [56] designed and synthesized a NiMn LDHs@Ni_3_S_2_ nanorod array on Ni foam via a hydrothermal method. The as-prepared hybrid arrays as electrode of SC exhibited a specific capacitance of 2703 F g^−1^ at 2 A g^−1^**.** Lin and co-workers [57] reported a hybrid structure consisting of CuCo_2_S_4_ nanorod as core and NiMn LDH as shell which was in situ synthesized by hydrothermal on Ni foam. The CuCo_2_S_4_@NiMn LDH core–shell array electrode showed a specific capacitance of 2520 F g^−1^ at 1 A g^−1^. 

**Figure 5:**
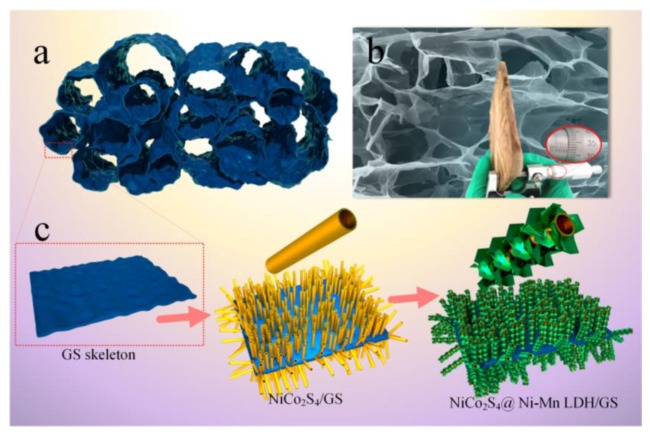
Process for the growth of 3D hierarchical structure: (**a**) the diagram for GO sponge; (**b**) SEM image and photo image (inserted in) of GO sponge; and (**c**) schematic illustration of the fabrication process of NiCo_2_S_4_ nanotube@NiMn LDH arrays. Reproduced with permission from [59]. Copyright ACS, 2015.

Bulky conductive carbon substrates are also commonly applied to deposit NiMn LDHs, similar to nickel foam. Li et al. [35] prepared an electrode material of flower-like NiMn LDH nanoarray that was grown on melamine sponge-derived carbon network for SC. The well dispersed NiMn LDH nanosheets resulted in the improved accessibility of active metal sites, more efficient Faradaic reactions and large stored-charge capacity. Honeycomb-like NiMn LDHs nanostructures were also grown on carbon cloth substrate via a hydrothermal method, showing high electrochemical performances [58]. Jiang group first prepared a 3D NiCo_2_S_4_ nanotube@NiMn LDH hybrid array in situ on graphene sponge network architectures, where the graphene sponge was obtained from a colloidal suspension of GO by a freeze-drying technique [59]. The highly conductive NiCo_2_S_4_ film showed excellent pseudocapacity and conductive support for high-performance NiMn LDH. The process for the growth of the 3D hierarchical configuration is shown in Figure 5.

**Figure 6:**
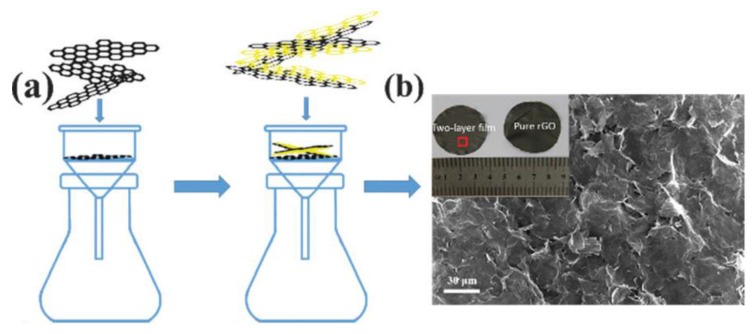
(**a**) Fabrication of the electrode by filtration method; and (**b**) top-view SEM image of the film electrode, inset showing a composite film and pure rGO paper. Reproduced with permission from [60]. Copyright Royal Society of Chemistry, 2016.

The above results show that, after the deposition of NiMn LDHs onto the surface of 3D skeleton conductive substrate, the formed core/shell materials can deliver a rather high specific capacitance. In addition to deposition of NiMn LDH onto conductive substrates, the other method to prepare NiMn LDHs electrode without organic binder is constructing a free-standing NiMn LDH film electrode. Free-standing films of NiMn LDH/graphene with a superlattice structure were reported by Quan et al. [60]. They designed a preparation method of film electrode via a filtration process with an rGO paper as the substrate and NiMn LDH/graphene composite as the functional film. The free-standing film electrode exhibited pseudocapacitive behavior and good rate capability owing to the nanosized effects. The fabrication process is schematically shown in Figure 6. Such a unique structure has ultralight weight of free-standing rGO paper and high performance of NiMn LDH/graphene.

### 3.5. Chemical Modification of NiMn LDHs Composites

Compared with bi-active metal hydroxides, i.e., NiMn LDH, multi-active metal hydroxides have a higher utilization of the electroactive centers due to the homogeneous elemental dispersion. Thus, the chemical composition of NiMn LDH host layers can be changed to realize an enhanced performance. NiCoMn-triple hydroxide was thus reported as a potential electrode material for SC application. Pure NiCoMn LDH and 3D NiCoMn LDH/rGO composites were synthesized by a simple solution method, as reported by Li et al. [61]. Electrochemical measurements proved that the incorporation of rGO could remarkably improve its electrochemical performances, compared to the NiCoMn LDH counterpart. A high specific capacitance of 912 F g^−1^, high rate capability as well as long life were achieved for the NiCoMn LDH/rGO material. At the same time, Xiong [62] prepared NiCoMn triple hydroxide nanoneedles that were coated on plasma-grown graphitic petals for pseudocapacitive electrodes. Comparing the electrochemical performance of the 3D NiCoMn LDH to NiCo LDHs revealed that a synergistic effect of the structure of graphitic petals and hydroxide enabled their high rate capability and long cycle life. Singh et al. [39] also reported that cobalt-doping for NiMn LDH was an effective approach to promote the electrochemical performance.

Very recently, Zhao et al. [28] applied density functional theory calculation to investigate a series of nickel hydroxides in basic media, typically for NiCoMn LDHs. Their results showed that Mn substitution mainly contributed to the enhancement of capacity due to the low deprotonation energy and easy electron transport, while cobalt substitution could chiefly stabilize the structure [28]. The existence of Mn in the host layer of NiMn LDH could also improve the electrocatalytic properties than pure nickel hydroxide. It was essentially associated to specific Mn sites which showed the lowest deprotonation energy that was related to high electron mobility, as reported by Oliver-Tolentino et al. [63]. Zheng et al. assembled NiCoMn LDH nanosheets hollow cages from bi-metallic imidazolate frameworks as precursor [64]. The ternary NiCoMn LDH hollow cages, as an electrode material for supercapacitor, demonstrated a remarkable performance and had a high specific capacitance of 2012.5 F g^−1^ at 1A g^−1^ in 1 M KOH. The above reports have demonstrated that the modification by some guest metals, such as cobalt in the lattice of NiMn LDH, can enhance the electrochemical performance and stability to some extent.

Table 1 summarizes the electrochemical performances of NiMn LDHs electrodes published in recent years. Regarding the specific capacitance, we can see that the values are dispersive in a wide range. It should be caused by the different NiMn LDH composition, inconsistent mass loading on the electrode, various contents of components in the composites. However, all these values are actually very high, typically for the NiMn LDH composites and films, showing their potential application for SCs.

### 3.6. NiMn LDH Materials for Asymmetric Capacitor

According to the assembly configurations of SCs, there are two kinds of SCs, symmetric and asymmetric (or hybrid) SCs. Designing an asymmetric SC is an efficient strategy to further improve the energy density via enlarging the potential window, because the energy density of a SC is generally in proportion to the square of potential value. Asymmetric (or hybrid) devices are thus regarded as a new developing trend for SCs.

Asymmetric capacitor, also called hybrid SC or battery capacitor, has been developed accompanying with the electrode materials [66,67]. An asymmetric capacitor usually consists of two dissimilar electrodes, i.e., a Faradaic positive electrode and an EDLC negative electrode, an insulative separator and electrolyte. Being compared to symmetric SCs, asymmetric SCs can take advantages of the two different voltage windows from the both electrodes, thus leading to a wide working voltage, even up to 2 V with aqueous electrolytes [68]. Carbon materials, typically activated carbon (AC), graphene and rGO, are commonly applied as a negative electrode due to their abundant sources, high surface area, high conductivity, fast charging/discharging speed and suitable voltage windows at negative potential [69]. These electrode materials that are determined by Faradaic redox reactions, such as metal oxides/hydroxides [70,71], metal sulfides [72], and conductive polymers [73], are normally used as a positive electrode for asymmetric SCs due to the high specific capacitance. In this section, we briefly review the reports related to asymmetric SCs using NiMn LDHs-based electrode.

**Figure 7:**
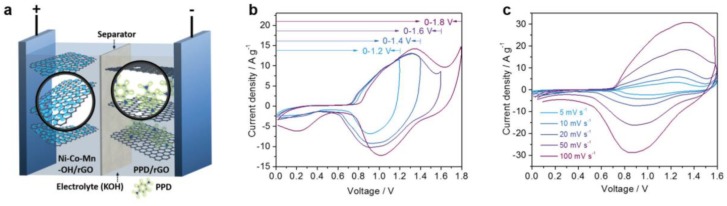
(**a**) Schematic illustration for a hybrid SC device with NiCoMn-OH/rGO electrode (+) and PPD-modified rGO electrode (−); (**b**) CV curves at the rate of 30 mV s^−1^ under various voltage windows; and (**c**) CV curves at 5–100 mV s^−1^. Reproduced with permission from [28]. Copyright Wiley, 2018.

A case of asymmetric SC study is shown as following. Figure 7a schematically shows an asymmetric SC, where NiCoMn hydroxide/rGO composite is employed as a battery-type electrode and organic molecular-modified (PPD) rGO is a capacitive electrode [28]. The possible working voltage of the asymmetric SC was tested by cyclic voltammetry from 1.2 to 1.8 V, as shown in Figure 7b. An optimized voltage of 0–1.6 V was thus determined from the curves. A further increasing voltage to 1.8 V would result in a dramatic increase in current density due to electrolyte decomposition or water splitting. The rate capability of the device was also evaluated by cyclic voltammetry at various scan rates, as shown in Figure 7c. The broaden redox peaks can be attributed to the combination of a capacitive electrode and a battery-like electrode. When the scan rate was increased from 5 to 100 mV s^−1^, the general shape of the CV curves was well maintained. Thus, under an asymmetric configuration, the operating voltage of hybrid SCs is dramatically enlarged.

For asymmetric SC device, the charge balance of positive and negative electrodes is essential for the electrochemical properties. The charge balance theory, i.e., *Q*^+^ = *Q*^−^, is a principle to judge the best match degree of the positive and negative electrodes. Thus, the mass of the electrode materials in the two electrodes should be balanced before assembly. Different from the electrode materials measured in a three-electrode system, asymmetric SC is much more similar to a battery and should be measured in a two-electrode assembly. In addition, to keep the high powder density and long service life of asymmetric SCs, it is also necessary to improve the energy density of asymmetric SCs. Powder density, energy density and operating voltage are three important parameters for asymmetric SCs. In Table 2, some parameters of asymmetric SCs are summarized and compared straightforward. We can see that the asymmetric SCs based on both NiMn LDH and carbon electrodes can deliver robust performances from the high values of energy density. The operating voltage of the asymmetric SCs is higher than 1.5 V. However, further work on the NiMn LDHs-based asymmetric SCs is also necessary.

## 4. Summary and Outlook

To obtain a high-performance SC, the electrode material should have the following features: high theoretical specific capacitance, high electrical conductivity, excellent cycling stability, enlarged potential window, low toxicity, and low cost [74,75]. NiMn LDHs have most of the above features. However, the low conductivity of NiMn LDH limits the capacitive performance and cycling stability severely. Although we can design rational nanostructures during the synthesis process to improve the electrochemical performance of NiMn LDHs, the complexity of the preparation procedure will hinder the practical application. However, NiMn LDH composites and thin films grown on conductive substrates including nickel foam and carbon cloth show researchers some clues to industrial application. The asymmetric SCs based on NiMn LDHs electrode show high energy densities with wide working voltages.

NiMn LDHs direct application as electrode materials of SCs represents a potential approach for energy storage. Recently, ternary metal oxides that contain two different transition metal oxides have also obtained popularity as electrode materials of SCs. Thus, NiMn LDH can be an ideal precursor for the preparation of ternary metal oxides, such as NiO and MnO_X_, showing wide application for SCs and Li ion batteries. Chen et al. [76] reported that NiMn layered double oxides electrode materials prepared through a carbonization process using NiMn LDH exhibited excellent performance and a high specific capacitance of 1648 F g^−1^ at 0.5 A g^−1^ in 1 M KOH**.** Latorre-Sanchez et al. [32] annealed NiMn LDH/GO composite at 450 °C under an inert atmosphere, leading to Ni_6_MnO_8_ nanoparticles dispersed on the surface of reconstituted graphene nanosheets after calcination. The composite material exhibited the electrical conductivity similar to graphite and it could be used as an anode for Li-ion batteries. It could deliver a maximum capacity of 1030 mAh g^−1^. The Ni-Mn oxide nanoparticles derived from NiMn LDHs could be also used as a potential catalyst for oxygen evolution reaction [77]. Thus, NiMn LDH materials have another great potential as the precursors of electrode materials for Li ion batteries, fuel cells and electrocatalysts.

In addition to using NiMn LDHs to prepare metal oxide, it can be even transformed into metal sulfides by simple sulfidation. These metal sulfides containing Ni and Mn have been reported as potential materials for SCs. Lee [65] group developed a strategy to prepare NiMn LDH by sulfidation to reduce the charge transfer resistance of SC electrode. The incorporation of GO in NiMn LDH during the sulfidation process leaded to simultaneous reduction of GO and S doping into the graphitic layers. The sulfidation product from NiMn LDH/GO could deliver a higher specific capacitance than both NiMn LDH and NiMn LDH/GO [65]. This work showed readers one more method to boost NiMn LDH materials, because metal sulfide usually exhibited a higher conductivity than the counterpart hydroxide [78]. Thus, sulfidation of NiMn LDH material can further reduce its resistance to realize a high performance. All aforementioned strategies and treatment methods show us the very promising prospects of NiMn LDHs for SC application.

## Figures and Tables

**Table 1:** Summary of the performances of NiMn LDH electrode materials.

Method	Materials	Electrolyte	Specific Capacitance (F g^−1^)	Current Density (A g^−1^)	References
Precipitation	Ni Mn LDH/CNTs/rGO	2 M KOH	1268	1	[23]
Precipitation	Ni-Mn LDHs hollow spheres	2 M KOH	595.6	1	[33]
Reverse micelle	NiMn LDH nanosheets	1 M KOH	881	1	[40]
Precipitation	NiMn LDHs/halloysite	2 M KOH	1665	1	[25]
Hydrothermal	Flower-like NiMn LDH/rGO	6 M KOH	1500	1	[37]
Precipitation	NiMn-LDH/rGO	3 M KOH	1250	1	[49]
Precipitation	NiMn-LDH/rGO	2 M KOH	1635	1	[24]
Hydrothermal	NiMn LDH/graphene	2 M KOH	2219	0.73	[50]
Precipitation	NiMn LDH/CNTs	1M KOH	2960	1	[48]
Hydrothermal	Glucose intercalated NiMn LDH	6 M KOH	1464	0.5	[44]
Precipitation	NiMn LDH/MnO_2_ nanosheets	1 M KOH	380	10 m v^−1^	[47]
Hydrothermal	Polyhedral NiMn LDH@carbon	6 M KOH	1634	1	[51]
Precipitation	NiMn LDH/rGO	2 M KOH	1209	2	[28]
Hydrothermal	NiMn LDH/GO	2 M KOH	1188	1	[65]
Precipitation	NiMn LDH@Ni foam	1 M KOH	1511	2.5	[52]
Hydrothermal	NiO/NiMn LDH@Ni foam	3 M KOH	937	0.5	[53]
Hydrothermal	Carbon coated NiMn LDH @Ni foam	1 M KOH	1863	1	[36]
Hydrothermal	Free-standing NiMn LDH/rGO	1 M KOH	421	36	[60]
Precipitation	KCu_7_S_4_/NiMn LDHs@Ni foam	1 M LiOH	773.8	1	[30]
Hydrothermal	NiCo_2_S_4_/NiMn LDH@graphene sponge	6 M KOH	1740 mF cm^−2^	1 mA cm^−2^	[59]
Hydrothermal	CuCo_2_S_4_/NiMn LDH@Ni foam	6 M KOH	2250	2	[57]
Hydrothermal	Ni_3_S_2_/NiMn LDH@Ni foam	1 M KOH	2703	3	[56]
Hydrothermal	LDH/vertical graphene@Ni foam	6 M KOH	2920	2	[55]
Precipitation	NiCoMn LDH/rGO	2 M KOH	912	1	[61]
Hydrothermal	Carbon/NiCoMn LDH@carbon cloth	2 M KOH	1400	0.5 mA cm^−2^	[62]
Hydrothermal	NiCoMn LDH (10% Co)	3 M KOH	2420	1	[39]
Precipitation	NiCoMn LDH nanocages	1 M KOH.	2012.5	1	[64]

**Table 2:** Summary of the asymmetric SCs based on NiMn LDH and carbon electrodes.

Negative Electrode	Positive Electrode	Power Density (kW kg^−1^)	Energy Density (Wh kg^−1^)	Potential (V)	References
rGO	Flower-like NiMn LDH/rGO	0.4	29.3	1.6	[37]
rGO	NiMn-LDH/rGO	1	22.5	1.8	[49]
AC	NiMn-LDH/rGO	0.85	33.8	1.7	[24]
rGO/CNT	NiMn LDH/CNTs	0.85	88.3	1.7	[48]
AC	Polyhedral NiMn LDH@carbon	0.225	18.6	1.5	[51]
rGO	NiMn-LDH/rGO	1.68	74.7	1.6	[28]
AC	NiO/NiMn LDH@Ni foam	0.41	27.8	1.6	[53]
AC	Carbon coated NiMn LDH @Ni foam	0.378	37.7	1.5	[36]
Graphene	KCu_7_S_4_/NiMn LDHs@Ni foam	0.65	15.9	1.5	[30]
AC	CuCo_2_S_4_/NiMn LDH@Ni foam	1.499	45.8	1.5	[57]
AC	Ni_3_S_2_/NiMn LDH@Ni foam	0.6	68	1.6	[56]
AC	LDH/vertical graphene@Ni foam	0.26	56.8	1.6	[55]
AC	NiCoMn LDH/rGO	4.24	42.4	1.8	[61]
rGO	NiCoMn-(LDH) (10% Co)	0.75	57.4	1.5	[39]
